# Applying machine learning to international drug monitoring: classifying cannabis resin collected in Europe using cannabinoid concentrations

**DOI:** 10.1007/s00406-024-01816-w

**Published:** 2024-05-21

**Authors:** Tom P. Freeman, Edward Beeching, Sam Craft, Marta Di Forti, Giampietro Frison, Christian Lindholst, Pieter E. Oomen, David Potter, Sander Rigter, Kristine Rømer Thomsen, Luca Zamengo, Andrew Cunningham, Teodora Groshkova, Roumen Sedefov

**Affiliations:** 1 Addiction and Mental Health Group (AIM), Department of Psychology, University of Bath, Bath, UK; 2 Hugging Face, Paris, France; 3 Department of Social, Genetic and Developmental Psychiatry, Institute of Psychiatry, Psychology and Neuroscience, King’s College London, London, UK; 4 Laboratory of Clinical and Forensic Toxicology, DMPO Department, AULSS 3, Venice, Italy; 5 Section for Toxicology and Drug Analysis, Department of Forensic Medicine, Aarhus University, Aarhus, Denmark; 6 Trimbos Institute, The Netherlands Institute of Mental Health and Addiction, Utrecht, Netherlands; 7 Canterbury, UK; 8 Centre for Alcohol and Drug Research, Department of Psychology and Behavioural Sciences, Aarhus University, Aarhus, Denmark; 9 European Monitoring Centre for Drugs and Drug Addiction (EMCDDA), Lisbon, Portugal; 10 Cannabis Clinic for Psychosis, South London and Maudsley Foundation Trust, London, UK

**Keywords:** Delta-9-tetrahydrocannabinol, Cannabidiol, Drug policy, Psychiatric disorders

## Abstract

In Europe, concentrations of ∆^9^-tetrahydrocannabinol (THC) in cannabis resin (also known as hash) have risen markedly in the past decade, potentially increasing risks of mental health disorders. Current approaches to international drug monitoring cannot distinguish between different types of cannabis resin which may have contrasting health effects due to THC and cannabidiol (CBD) content. Here, we compared concentrations of THC and CBD in different types of cannabis resin collected in Europe (either Moroccan-type, or Dutch-type). We then tested the ability of machine learning algorithms to classify the type of cannabis resin (either Moroccan-type, or Dutch-type) using routinely collected monitoring data on THC and CBD. Finally, we applied the optimal algorithm to new samples collected in countries where the type of cannabis resin was unknown, the UK and Denmark. Results showed that overall, Dutch-type samples had higher THC (Hedges’ g = 2.39) and lower CBD (Hedges’ g = 0.81) than Moroccan-type samples. A Support Vector Machine algorithm achieved classification accuracy exceeding 95%, with little variation in this estimate, good interpretability, and plausibility. It made contrasting predictions about the type of cannabis resin collected in the UK (94% Moroccan-type; 6% Dutch-type) and Denmark (36% Moroccan-type; 64% Dutch-type). In conclusion, we provide proof-of-concept evidence for the potential of machine learning to inform international drug monitoring. Our findings should not be interpreted as objective confirmatory evidence but suggest that Dutch-type cannabis resin has higher THC concentrations than Moroccan-type cannabis resin, which may contribute to variation in drug markets and health outcomes for people who use cannabis in Europe.

## Introduction

In the past decade, data from the European Monitoring Centre for Drugs and Drug Addiction (EMCDDA) have documented a marked increase in ∆^9^-tetrahydrocannabinol (THC) concentrations of cannabis resin samples collected in Europe [1–3]. Such trends have also been reported in country-specific studies of cannabis resin in Europe [4] such as in France [5], Italy [6] and Denmark [7]. These developments are important because use of cannabis with higher THC concentrations is associated with an increased risk of mental health disorders [8], in particular cannabis use disorder [9, 10] and psychotic disorder [11, 12]. Therefore, a better understanding of the European cannabis resin market is critical for assessing the potential health effects of cannabis in Europe, and to inform international policies to reduce harms.

The reasons for increasing THC concentrations in cannabis resin collected in Europe are currently unclear. However, they could potentially be attributable to changes in the prevalence of different types of cannabis resin with varying cannabinoid concentrations. Morocco is believed to be the single largest producer of cannabis resin trafficked to Europe. One potential contributor to increases in THC concentrations in cannabis resin collected in Europe is changes to Moroccan cannabis resin production methods. For example, it has been suggested that lower potency traditional Moroccan ‘kif’ plants have been replaced with high-THC hybrid strains of cannabis, increasing THC concentrations in Moroccan-type resin [13]. This is corroborated by forensic analysis reporting increases in THC concentrations in cannabis samples collected in Morocco [14]. However, some evidence suggests differences between cannabis resin sampled from Europe and Morocco. For example, cannabis resin samples have had higher THC concentrations when collected in some European countries such as Denmark [7] when compared to cannabis resin collected in the same time frame in Morocco [14]. Indeed, cannabis resin collected in Denmark showed the highest THC concentrations of all European countries submitting data to the EMCDDA [2]. Moreover, Moroccan cannabis resin trafficked to Europe (for export) may differ from that sampled from Morocco (for domestic use).

**Fig. 1:**
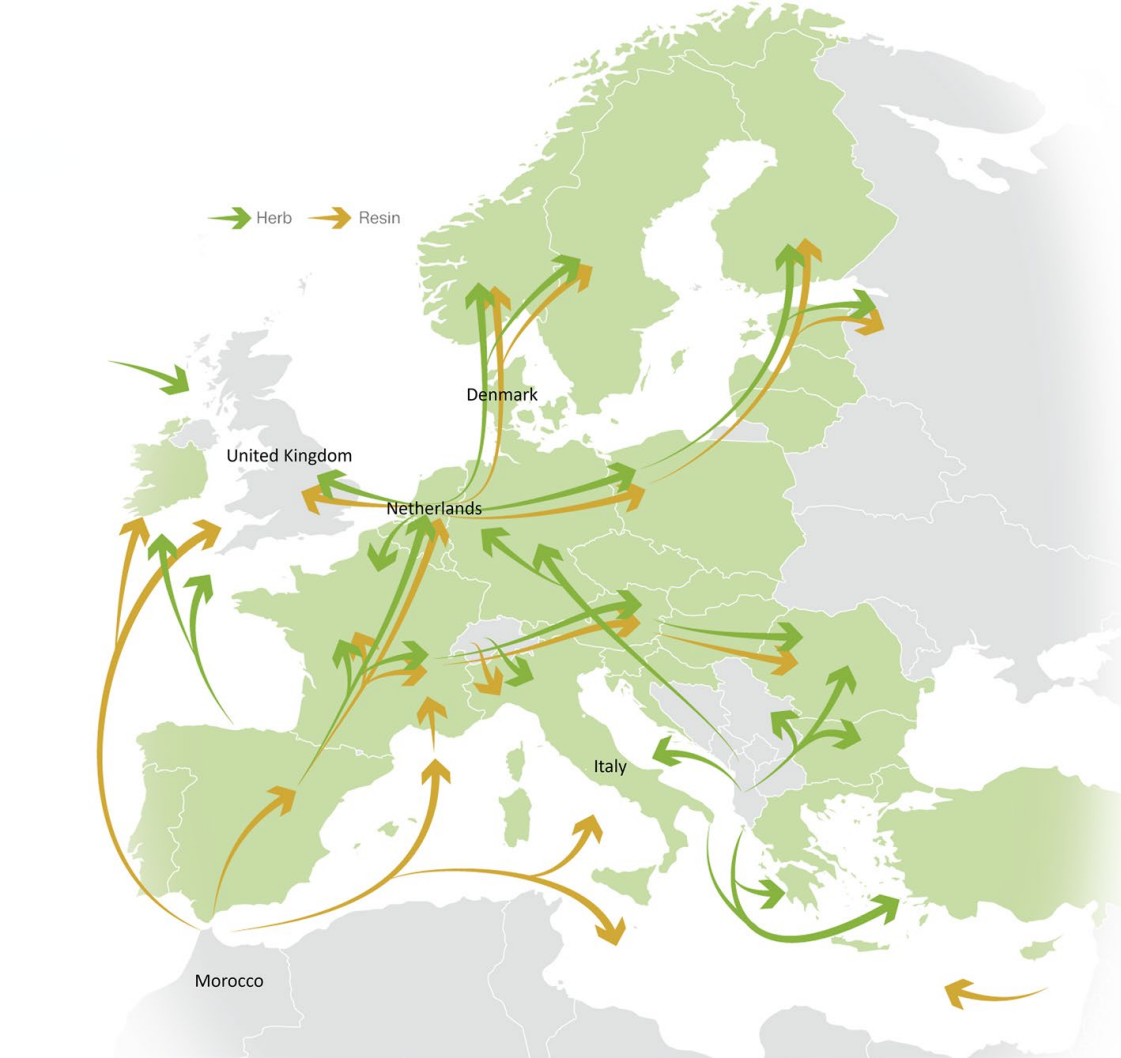
Trafficking routes for cannabis products in Europe. Cannabis resin is trafficked from Morocco through Europe. The Netherlands is a major hub for trafficking cannabis resin to other European countries such as the United Kingdom and Denmark. Figure adapted from EMCDDA-Europol EU Drug Markets Report [1]

Another possible explanation for increasing THC concentrations in cannabis resin collected in Europe is domestically produced cannabis resin [15]. In the Netherlands, cannabis resin has been produced from domestic herbal cannabis for many years as a distinct product called ‘Nederhasj’ [16]. Nederhasj can be produced using ice-based extraction or a ‘pollinator’, which are more efficient at extracting THC-containing trichomes from cannabis plant material than traditional extraction methods such as sieving. Additionally, production of cannabis plants using particular strains and indoor growing conditions can increase levels of THC in the cannabis plant [17]. Importantly, the type of plant material used for extraction will influence the final product in terms of THC/CBD profile. Given that domestically produced herbal cannabis in Europe is the primary product used for extraction, it can be expected that the majority of domestically produced European cannabis resin will also primarily contain THC-dominant cannabis plant material, resulting in a greater concentration of THC and higher price per gram of cannabis [2]. The Netherlands is a major hub for production and trafficking of cannabis resin within Europe (Fig. 1), and Dutch-type cannabis resin may potentially have contributed to increases in THC concentrations in cannabis resin reported at the European level and in individual countries.

At present there are no methods available to help identify the type of cannabis resin collected in Europe. This could give an indication of potential health effects [based on concentrations of THC and other cannabinoids such as cannabidiol (CBD [18])], plant source and production method, to inform international drug monitoring (e.g., possible trafficking routes in Europe and neighbouring countries; Fig. 1) and policies to improve health and security. Here we aimed to classify cannabis resin samples collected in Europe into two types based on plant source and production methods associated with two key countries (Moroccan-type and Dutch-type). If supported by accurate classification, this tool may give an indication of the type of cannabis resin collected in new unclassified samples, including the type of cannabis plant material used prior to extraction (including relative concentrations of THC and CBD) as well as the efficiency of the extraction process. Here, we use the terms “Moroccan-type” and “Dutch-type” to refer to the plant source and production method associated with these two countries, but this should not be interpreted as definitive evidence of sample origin.

The objectives of this study were to (1) compare concentrations of THC and CBD in different types of cannabis resin samples collected in Europe (Moroccan-type or Dutch-type), (2) test the ability of machine learning algorithms to classify the type of cannabis resin (Moroccan-type or Dutch-type) based on THC and CBD concentrations, and (3) to apply the optimal algorithm to new cannabis resin samples collected in countries where the type of sample is unknown (the UK and Denmark). Due to the novelty and exploratory nature of this study we did not intend to generate objective confirmatory evidence, but instead to demonstrate proof-of-concept for the potential of using machine learning to inform international drug monitoring. By identifying the potential health effects of different types of cannabis resin due to their cannabinoid concentrations (objective 1) and developing a novel tool to enable identification of cannabis resin samples in Europe (objectives 1 and 2) this approach could inform novel approaches to international drug monitoring that could be applied at the individual drug sample level, as well as at the country- and European- level.

## Methods

This study applied statistical methods to routinely collected monitoring data on cannabis resin samples collected in Europe. It was conducted as part of the EU4Monitoring Drugs project, an EMCDDA project funded by the European Union to establish links between drug-related problems and security and health threats in the European Union and neighbouring countries.

### Moroccan-type cannabis resin

We searched for studies reporting on cannabinoid concentrations in Moroccan-type cannabis from 2015 onwards, to coincide with recent increases in THC concentrations in cannabis resin collected in Europe [1–3]. The authors of studies reporting on Moroccan-type cannabis resin prior to 2015 [14, 19] were contacted to seek more recent data, but none were available. The only study providing relevant data was Zamengo et al. [6] who analysed cannabinoid concentrations from cannabis resin samples collected in North-East Italy. This study included cannabis samples seized in relation to drug dealing offences rather than possessions of small quantities (and therefore would be of greater chance of being related to trafficked products). Importantly, individual products were linked to the people involved in the seizure; therefore, in this study we restricted analysis data to those samples linked to Morocco (and no other countries) and collected from 2015 to 2019. Because these samples were collected in Europe and linked to Morocco at the individual sample level, they may offer a better indicator of cannabis resin trafficked from Morocco to Europe than cannabis resin samples collected in Morocco. Principal Components Analysis of cannabinoid concentrations in that study [6] offered some validation for the differentiation between different cannabis product types based on sample origin.

### Dutch-type cannabis resin

The Netherlands is the only European country that conducts repeat cross-sectional cannabis monitoring using randomised sampling. A random selection of national cannabis retail outlets are selected for purchases in January each year to control for seasonal variation [20]. This standardised purchasing protocol includes Nederhasj, which is produced by extracting cannabinoids using ice or pollinator methods from herbal cannabis grown in the Netherlands. For the purposes of this study, the results of the analysis of Nederhasj samples purchased over a five-year period (from 2015 to 2019) were obtained to represent cannabis resin produced in the Netherlands. Nederhasj samples were used because they are produced domestically in the Netherlands and thus are not imported from other countries such as Morocco. Such samples were distinguished from imported products based on the product description at the point of sale.

### Classification of samples as Moroccan- or Dutch- type

To classify cannabis resin samples as Moroccan- or Dutch- type using routinely collected monitoring data, sample-level concentrations of THC and CBD were obtained. Firstly, the Moroccan- or Dutch- type samples were compared at the group level for concentrations of THC and CBD using 1000 bootstrapped samples with bias-corrected accelerated 95% confidence intervals. Effect sizes for group level comparisons were estimated using Hedges’ g (small effect size = 0.2, medium effect size = 0.5, large effect size = 0.8). Next, a series of machine learning algorithms were tested for their ability to classify individual samples as being Moroccan- or Dutch- type using these data. The methods used were as follows:

### Logistic regression [21]

Logistic regression is a statistical method used for binary classification tasks, extending from linear regression by applying a sigmoid function to the predictions, so that outputs are probabilities that a given input belongs to a particular class.

### K-Nearest Neighbours [22]

K-Nearest Neighbours is a simple learning algorithm where the class of a sample is determined by the majority class among its k-nearest neighbours. K-nearest neighbours is non-parametric and lazy, meaning it does not explicitly learn a model, but rather memorises the training dataset, making it versatile but also computationally intensive for large datasets.

### Decision Tree [23]

A Decision Tree is a flowchart-like tree structure where internal nodes represent tests on attributes, branches represent the outcome of these tests, and leaf nodes represent class labels or regression values. It is a simple yet powerful tool for classification and regression tasks, offering models that are easy to interpret but prone to overfitting.

### Random Forest [24]

Random Forest is an ensemble learning method that operates by constructing multiple decision trees at training time and outputting the class that is the mode of the classes (classification) or mean prediction (regression) of the individual trees. It improves upon the decision tree method by reducing overfitting and increasing prediction accuracy through ensemble averaging.

### Support Vector Machine [25]

Support Vector Machines are a set of supervised learning methods used for classification, regression, and outlier detection. The core idea of Support Vector Machines is to find the hyperplane that best divides a dataset into classes, with the goal of maximizing the margin between data points of different classes. Support Vector Machines are effective in high-dimensional spaces and versatile, due to different kernel functions that can be specified for the decision function.

### Naïve Bayes [26]

Naive Bayes classifiers are a family of simple probabilistic classifiers based on applying Bayes' theorem with strong (naive) independent assumptions between the features. Despite its simplicity, Naive Bayes classifiers work well in many real-world situations, notably document classification and spam filtering. They are particularly efficient in scenarios where the dimensionality of the inputs is high relative to the amount of data.

Each of these methods were tested using K-fold cross validation [27], with five training/test iterations. Each iteration consisted of a random sample of 80% data for training the algorithm, and 20% of the data to test its accuracy. We explored the impact of feature engineering through inclusion of square/log of the predictors on the performance of the machine learning algorithm. We did not observe an improvement in cross-validation performance and did not include these features in the final models to avoid overfitting. The final performance of each of the models was assessed according to the mean accuracy scores and the standard deviation for each of these five iterations, to ensure that results were robust to bias from potential outliers. The optimal algorithm was chosen based on accuracy, variation in accuracy, interpretability, and plausibility.

### Classification of cannabis resin samples of unknown type

The optimal algorithm was then used to classify the type of cannabis resin collected in European countries where there was uncertainty about cannabis resin type—the UK and Denmark. Cannabis resin samples seized in the UK from 2015–2016 were obtained from a previous study including data from six constabularies in England: Derbyshire, Kent, London Metropolitan, Merseyside, and Sussex [28]. Cannabis resin samples seized in Denmark from 2015–2017 were obtained from a previous study, using samples collected from Aarhus in Western Denmark [7]. Additional data were provided through a request to the senior author of the Danish study (CL), resulting in a time frame of 2015–2018 available for this study.

## Results

### Moroccan- and Dutch- type cannabis resin

**Fig. 2:**
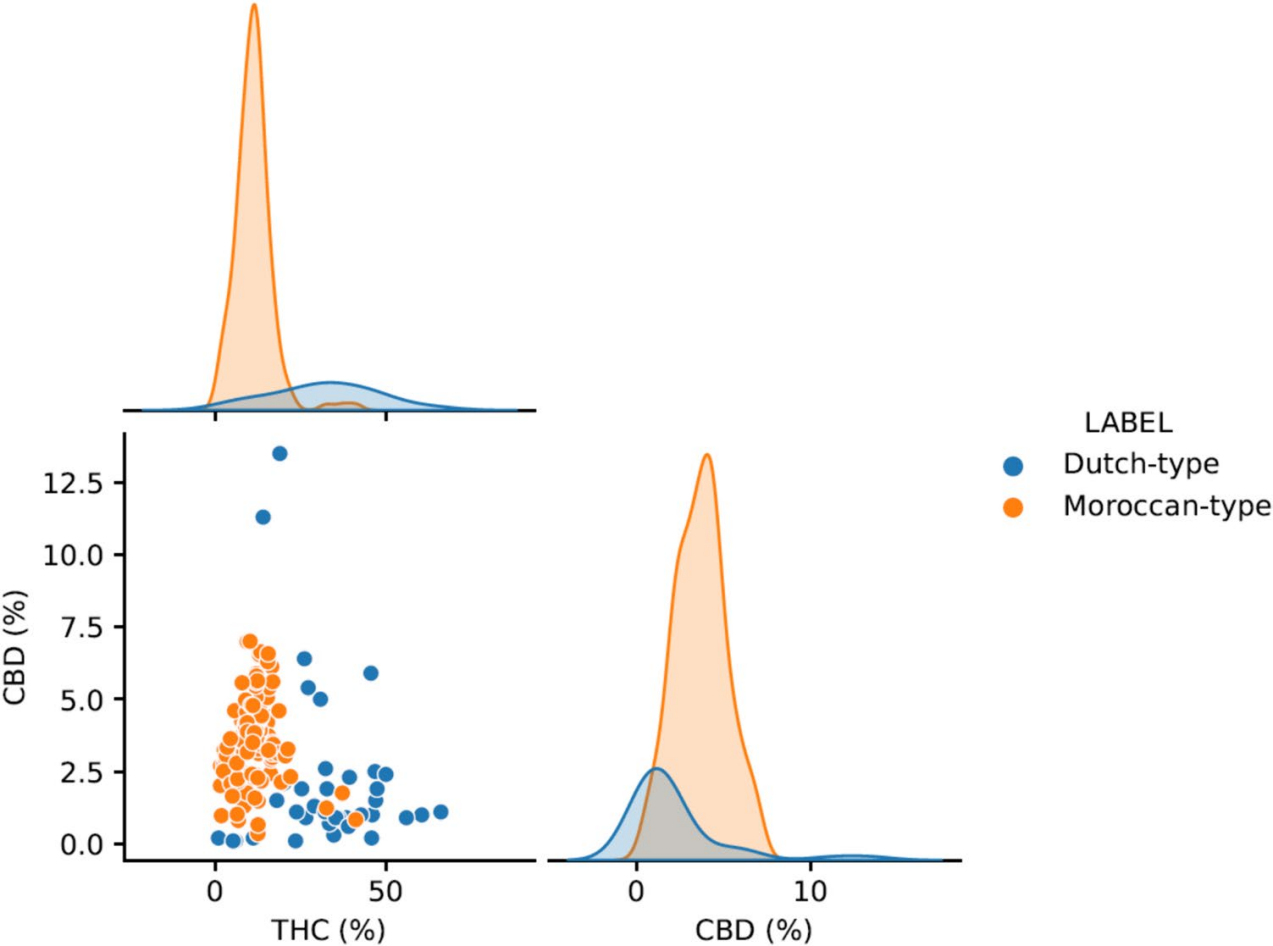
Plots showing delta-9-tetrahydrocannabinol (THC) and cannabidiol (CBD) concentrations in Dutch-type and Moroccan-type cannabis resin samples. As Gaussians are used to depict the distributions, these cross zero on the plots, but there are no raw datapoints below zero

Data were available for 189 cannabis resin samples, of which 40 were Dutch-type and 149 Moroccan-type. As shown in Fig. 2 and Table 1, THC concentrations were higher in Dutch-type samples than Moroccan-type samples with a mean difference of 20.55 (95% CI: 15.39, 25.54), p < 0.001, with a large effect size (Hedges’ g = 2.39). Additionally, CBD concentrations were lower in Dutch-type samples than Moroccan-type samples with a mean difference of −1.50 (95% CI −2.36, −0.47), p < 0.001, with a large effect size (Hedges’ g = 0.81). As shown in the scatterplot in Fig. 2, there was clear separation between Dutch-type and Moroccan-type cannabis resin samples according to their THC and CBD concentrations for most samples. However, there was greater variance around the mean for the Dutch-type samples for both THC and CBD concentrations, and particularly so for THC concentrations. There were also a limited number of Dutch-type samples with very high CBD concentrations, and of Moroccan-type samples with very high THC concentrations, which were outliers to this overall separation of distributions.

**Table 1:** Concentrations of delta-9-tetrahydrocannabinol (THC) and cannabidiol (CBD) in Dutch-type and Moroccan-type cannabis resin samples

		Mean	Median	SD	Minimum	Maximum
THC concentration (%)	Dutch-type	31.71	32.40	15.46	0.80	66.00
	Moroccan-type	11.16	11.20	5.52	1.40	41.00
CBD concentration (%)	Dutch-type	2.19	1.10	2.84	0.10	13.50
	Moroccan-type	3.69	3.70	1.47	0.40	7.00

### Classification of samples as Moroccan- or Dutch- type

**Fig. 3:**
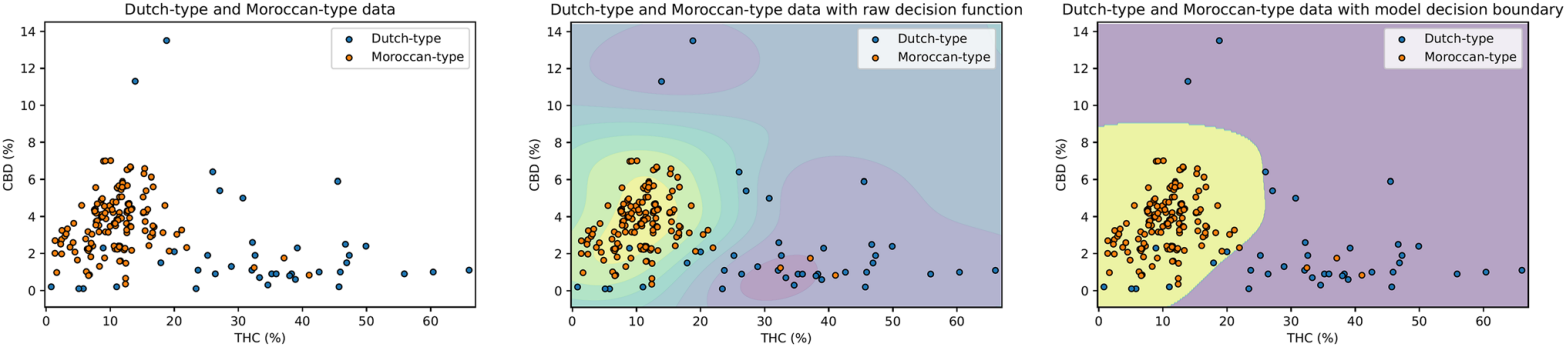
Plots showing delta-9-tetrahydrocannabinol (THC) and cannabidiol (CBD) concentrations in Dutch- and Moroccan- type cannabis resin (left) with raw decision function (middle) and decision boundary (right). The Support Vector Machine algorithm predicted that samples within the yellow region were Moroccan-type, and those within the purple region were Dutch-type. The accuracy of the model is indicated by the number of correctly identified Moroccan-type (orange dots in the yellow region) and Dutch-type samples (blue dots in the purple region)

As shown in Table 2, each of the machine learning algorithms showed high mean accuracy for classifying cannabis resin samples as either Moroccan- or Dutch- type based on THC and CBD concentrations. Two methods exceeded mean accuracies of 95%: Support Vector Machine and Naïve Bayes. Of those two methods, the Support Vector Machine algorithm was identified as the optimal method due to a lower standard deviation in the accuracy estimate. The performance of this model in successfully classifying the data is shown in Fig. 3, indicated by the decision boundary showing good interpretability and plausibility.

**Table 2:** Accuracy of machine learning algorithms in classifying cannabis resin samples as Dutch-type or Moroccan-type based on concentrations of delta-9-tetrahydrocannabinol (THC) and cannabidiol (CBD)

Method	Accuracy	
	Mean	SD
Logistic regression	0.933	0.026
K-nearest neighbour	0.942	0.028
Decision tree	0.944	0.023
Random forest	0.948	0.028
Support vector machine	0.952	0.016
Naïve bayes	0.954	0.020

### Classification of cannabis resin samples of unknown type

**Fig. 4:**
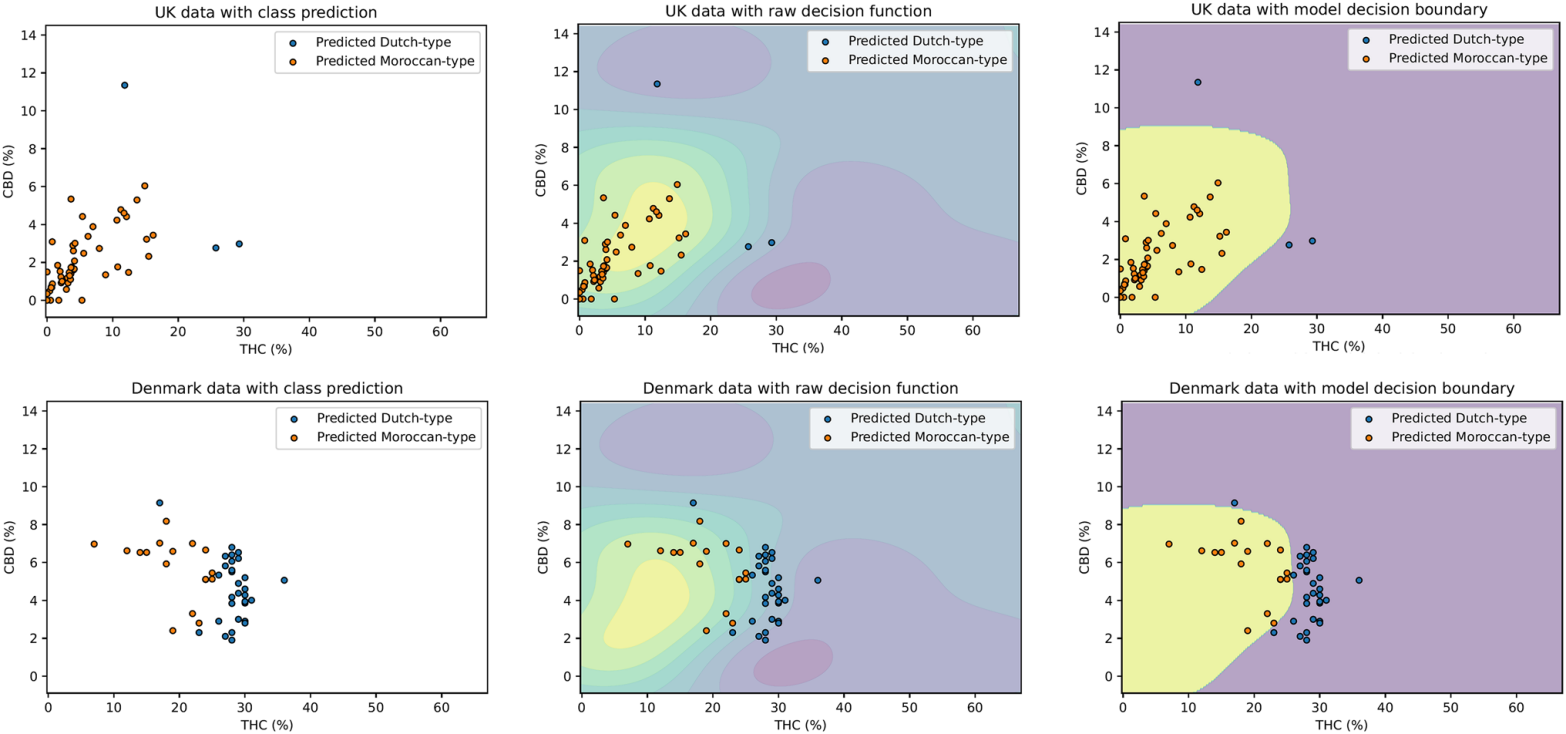
Plots showing delta-9-tetrahydrocannabinol (THC) and cannabidiol (CBD) concentrations in cannabis resin collected in the UK (top) and Denmark (bottom). Plots show data with class prediction (left), data with raw decision function (middle), and data with model decision boundary (right). The algorithm predicted that samples within the yellow region were Moroccan-type, and those within the purple region were Dutch-type

Next, the Support Vector Machine algorithm was applied to new data where the type of cannabis resin sample was uncertain: the UK and Denmark. A total of 101 samples were available, 54 from the UK and 47 from Denmark (see Table 3). As shown in Fig. 4, there were clear differences in predictions about the type of cannabis resin samples collected in these two countries. Of the 54 samples collected in the UK, 51 (94%) were classified as Moroccan-type and 3 (6%) as Dutch-type. By contrast, of the 47 samples collected in Denmark, only 17 (36%) were classified as Moroccan-type and 30 (64%) as Dutch-type (see Table 4).

**Table 3:** Concentrations of delta-9-tetrahydrocannabinol (THC) and cannabidiol (CBD) in cannabis resin samples collected in the UK and Denmark

		Mean	Median	SD	Minimum	Maximum
THC concentration (%)	UK	6.23	3.85	6.26	0.00	29.30
	Denmark	25.00	27.00	5.80	7.00	36.00
CBD concentration (%)	UK	2.29	1.69	1.97	0.00	11.35
	Denmark	5.01	5.10	1.73	1.90	9.10

**Table 4:** Concentrations of delta-9-tetrahydrocannabinol (THC) and cannabidiol (CBD) in cannabis resin samples collected in the UK and Denmark based on classification as Dutch- or Moroccan- type

	Country of collection	Support vector machine prediction	Mean	Median	SD	Minimum	Maximum
THC concentration (%)	UK	Dutch-type	22.29	25.74	9.23	11.84	29.30
		Moroccan-type	5.28	3.65	4.67	0.00	16.18
	Denmark	Dutch-type	28.23	28.00	3.00	17.00	36.00
		Moroccan-type	19.29	19.00	5.12	7.00	25.00
CBD concentration (%)	UK	Dutch-type	5.70	2.98	4.90	2.76	11.35
		Moroccan-type	2.09	1.60	1.55	0.00	6.04
	Denmark	Dutch-type	4.60	4.50	1.68	1.90	9.10
		Moroccan-type	5.72	6.50	1.61	2.40	8.20

## Discussion

Data from the EMCDDA have documented marked increases in THC concentrations in cannabis resin, raising concerns of mental health disorders for people who use cannabis in Europe. Here we report that Dutch-type cannabis resin collected in the Netherlands had higher THC concentrations and lower CBD concentrations than Moroccan-type cannabis resin collected in Italy during the same timeframe (2015–2019). We applied a series of machine learning algorithms to classify these samples as either Dutch- or Moroccan- type according to their THC and CBD concentrations. All algorithms tested showed a high level of accuracy and thus provided comparable performance. The optimal method was a Support Vector Machine algorithm, showing high accuracy (exceeding 95%), with low variance in this estimate, and good interpretability and plausibility. This algorithm was then used to classify new cannabis resin samples of unknown type collected in the UK and Denmark. Almost all samples seized in the UK were classified as Moroccan-type, whereas most samples seized in Denmark were classified as Dutch-type.

Overall, this study demonstrates considerable variation in cannabinoid concentrations, and therefore risk of health harms, of different types of cannabis resin collected in Europe. Moreover, it provides proof-of-concept evidence that machine learning has the potential to help identify cannabis resin samples of unknown type based on routinely collected monitoring data on cannabinoid concentrations. Although changes in Moroccan production methods have been cited as an explanation for rising THC concentrations in cannabis resin at the European level, the results of this study suggest that domestic European produced cannabis resin may have considerably higher THC concentrations than Moroccan-type cannabis resin, and may have plausibly contributed to rising THC concentrations in Europe [1–3] and particular countries such as Denmark [7]. This is consistent with a role of the Netherlands as a major cannabis resin producer and a trafficking hub for cannabis in Europe (including to Nordic countries such as Denmark) as shown in Fig. 1, although the methods we applied here are indicative of plant source and production method rather than a definitive test of sample origin. Rising THC concentrations in cannabis may increase the risk of health harms [8] including cannabis use disorder [9, 10] and psychotic disorder [11, 12]. International policies may benefit from considering the role of distinct cannabis resin production methods and plant source on variation in THC concentrations and health effects of cannabis use in Europe.

This study had some strengths. To our knowledge, this is the first study of its kind harnessing individual sample level data from multiple cannabis monitoring programmes in different countries. We used innovative methodology applying advanced mathematical methods to routinely collected data. Harnessing data from four distinct cannabis monitoring programmes and individual drug sample data linkage enabled us to address novel research questions with relevance for health and security in Europe and neighbouring countries. There are also some limitations of this study. The ability to classify samples accurately using machine learning is dependent on the quality of the data used to train the algorithm. The terms Moroccan-type and Dutch-type in this study may be indicative of the plant source and production method used, but do not necessarily indicate the location of production definitively. In ideal circumstances we would have had access to a wider range of data sources to confirm the type of cannabis resin. Yet because cannabis resin is an illegal product in most of Europe such data were unavailable. Certain factors may have contributed to a risk of bias, such as the modest sample sizes available. While the training dataset can be considered small (n = 189) for a machine learning algorithm, the choice of using only two predictors limited the capacity for the machine learning algorithm to overfit to the data. Although alternative (and simpler) approaches could have been used, our decision to test a range of machine learning algorithms increased our potential to identify a suitable approach based on accuracy, variation in accuracy, interpretability, and plausibility. Sample representativeness is an important consideration and can be considered adequate for Dutch-type data due to random sampling from national retail outlets. By contrast, sample representativeness is difficult to achieve when drug samples are obtained from law enforcement procedures, which is a ubiquitous problem in international drug monitoring. Although cannabis accounts for most illicit drug seizures in Europe there is a paucity of data from regular cannabis monitoring programmes in Europe and globally. Additionally, bias could have arisen from different subtypes of Moroccan-type and/or Dutch-type cannabis resin samples (e.g., such as the outlying values identified in the scatterplot), other types of cannabis resin in the European market, or the possibility that the type of cannabis resin used to train the algorithm was incorrect for some data points. Future research could benefit from harnessing larger sample sizes and data from a wider range of countries and products to improve the accuracy of training and classification. Larger sample sizes could also increase the ability to distinguish between the performance of different machine learning algorithms in future studies. Overall, this study provides proof-of-concept evidence for the value of using machine learning to inform international drug monitoring and should not be interpreted as objective confirmatory evidence. However, machine learning is a flexible and powerful approach that can readily incorporate new data to improve accuracy and account for new information.

In conclusion, we characterised differences in THC and CBD concentrations in different types of cannabis resin collected in Europe and used machine learning to classify the type of cannabis resin with over 95% accuracy. Application of this algorithm to cannabis resin samples collected in the UK and Denmark was suggestive of marked differences in the type of cannabis resin samples collected in different European countries. Almost all samples collected in the UK were classified as Moroccan-type, whereas most samples collected in Denmark were classified as Dutch-type. Taken together, these results suggest that plant source and production methods may be important contributors to variation in the European cannabis resin market, such as THC concentrations at the European and country-specific level (e.g., the UK versus Denmark). Classification of cannabis resin into different types could enhance international drug monitoring by helping to characterise changes in drug products, international trafficking routes, and to inform policies to improve health and security. The machine learning approach harnessed here could be applied to a range of other novel problems to improve international drug monitoring.

## Data Availability

For data requests, please contact the corresponding author.
